# 

**Published:** 1858-06

**Authors:** 


*Receipts on Medical Journal since last issue.*

| | | | |
|---|---|---|---|
| Dr. Collings, J. S. | $3 00 | Dr. Dorr, F. A. | $2 00 |
| Little, A. C. | 2 00 | Mallett, A. | 2 00 |
| Spees, —— | 6 00 | Stratton, F. M. | 2 00 |
| Williams, E. M. | 5 00 | | |

☞ Subscribers in arrears will confer a favor by forwarding the amount of their subscription. It must be evident to every one that the amount of receipts will not cover one-half the cost of publication ; we ask nothing for *our* labor, but we must *pay the printer.*

